# Accuracy of Three-Dimensional Soft-Tissue Prediction Considering the Facial Aesthetic Units Using a Virtual Planning System in Orthognathic Surgery

**DOI:** 10.3390/jpm12091379

**Published:** 2022-08-25

**Authors:** Daniel Awad, Siegmar Reinert, Susanne Kluba

**Affiliations:** Department of Oral and Maxillofacial Surgery, University Hospital Tübingen, Eberhard Karls University, Osianderstr. 2-8, 72076 Tübingen, Germany

**Keywords:** orthognathic surgery, soft-tissue prediction, virtual surgical planning

## Abstract

Virtual surgical planning (VSP) is commonly used in orthognathic surgery. A precise soft-tissue predictability would be a helpful tool, for determining the correct displacement distances of the maxilla and mandible. This study aims to evaluate the soft-tissue predictability of the VSP software IPS CaseDesigner^®^ (KLS Martin Group, Tuttlingen, Germany). Twenty patients were treated with bimaxillary surgery and were included in the study. The soft-tissue simulation, done by the VSP was exported as STL files in the engineering software Geomagic Control X^TM^ (3D systems, RockHill, SC, USA). Four months after surgery, a 3D face scan of every patient was performed and compared to the preoperative simulation. The quality of the soft-tissue simulation was validated with the help of a distance map. This distance map was calculated using the inter-surface distance algorithm between the preoperative simulation of the soft-tissue and the actual scan of the postoperative soft-tissue surface. The prediction of the cranial parts of the face (upper cheek, nose, upper lip) was more precise than the prediction of the lower areas (lower cheek, lower lip, chin). The percentage of correctly predicted soft-tissue for the face in total reached values from 69.4% to 96.0%. The VSP system IPS CaseDesigner^®^ (KLS Martin Group; Tuttlingen, Germany) predicts the patient’s post-surgical soft-tissue accurately. Still, this simulation has to be seen as an approximation, especially for the lower part of the face, and continuous improvement of the underlying algorithm is needed for further development.

## 1. Introduction

Today, three-dimensional (3D) virtual surgical planning (VSP) is a well-established procedure in orthognathic surgery, offering a precise simulation of the operative outcome. The facial skeleton and the surrounding soft-tissue are responsible for facial harmony and balance. The fundament on which the aesthetics of the face is based is formed by the architecture and topographic relationships of the facial skeleton. However, the visual impact of the face depends entirely on the form and proportion of the soft-tissues as claimed by Mandrekar et al. [1]. Most modern surgical planning programs provide the possibility not only of preoperative simulation of skeletal displacement but also of predicting the expected changes in the facial soft-tissue. For successful surgical planning, visualization of the proposed postoperative facial soft-tissue is important since the aesthetic outcome of elective surgery is typically one of the patient’s major concerns, and the availability of a realistic expectation of the outcome can enhance patient satisfaction [2,3,4].

The predictability of soft-tissue simulation has become one of the most prominent research subjects concerning orthognathic surgery since many surgeons and orthodontists shifted their attention from occlusion-based planning to soft-tissue based planning [5,6]. Moreover, VSP allows the surgeon to experience and assess different surgical scenarios. Different VSP programs with soft-tissue simulation tools are available for orthognathic surgery.

IPS CaseDesigner^®^ (KLS Martin Group; 78532 Tuttlingen, Germany) is among the well-established and commonly used VSP systems. In a recent study, Willinger et al. evaluated the soft-tissue predictability of this VSP software, but their analyses were limited to the case of intraoral quadrangular Le Fort II osteotomy [2]. To date, no study has evaluated the precision of this program’s soft-tissue predictability when bimaxillary orthognathic surgery is performed. Most studies analysed the soft-tissue predictability of VSP software by measuring different cephalometric landmarks, mostly for the chin and lower and upper lip regions [3,4,7,8,9,10]. Thereby, the measurement is restricted to a 2D perspective, although 3D VSP software was used. The purpose of this study was to examine the precision of the soft-tissue predictability of the VSP system IPS CaseDesigner^®^ by measuring the entire surface congruence between the actual postsurgical situation (3D-Scan) and the presurgical simulation after superimposition.

## 2. Materials and Methods

### 2.1. Patients

In total, 20 patients (10 female and 10 male) with skeletal class II or III malocclusion were included in this study. This study was conducted after receiving the ethics committee approval (Nr.: 717/2021BO2). All patients underwent orthognathic surgery from June 2019 to December 2021 at the Department of Oral and Maxillofacial Surgery, University Hospital Eberhard Karls University (Tübingen, Germany). The average age was 27.3 years at the time of surgery (range: 21–45). All patients were treated with bimaxillary surgery, including maxillary advancement by Le Fort I osteotomy and mandibular displacement by bilateral sagittal split osteotomy (BSSO), without any additional surgical procedures such as genioplasty, malar augmentation, or rhinoplasty. The inclusion criteria were a minimum age of 18 years and a minimum number of remaining teeth to ensure a sufficient occlusion, at least 13 occlusion units or more and very antagonizing pair of teeth was considered as one occlusion unit. Moreover, all patients had sufficient preoperative orthodontic treatment.

The exclusion criteria were a generally abnormal occlusion (for example, in cases of massive abrasions, large fillings with an insufficient reconstruction of the occlusal surface, or severe syndromic deformation); previous orthognathic surgery; or other orthognathic procedures, such as chin osteotomy. All included patients had no other diseases. For further examination, all patients were categorized by the following independent parameters: angle class (II or III); total displacement distance of the maxilla and/or mandible (<5 mm; >5 mm), initial anterior bite situation (deep = overbite > 2 mm; neutral = overbite 2–0 mm; open = overbite < 0 mm), age (<30 years, 30–40 years, >40 years) and gender (Table 1).

### 2.2. Data Acquisition

All patients received a cone beam computer tomography (CBCT) scan 3 weeks prior to surgery. The CBCT was performed using a KaVo 3D Orthopantomograph^TM^ (KaVo Dental^®^ GmbH; Biberach an der Riß, Germany) with the following parameters: 120 kVp; voxel size, 0.4 × 0.4 × 0.4 mm^3^; scan time 40 s; and field of view 22 × 16 cm^2^. All patients were scanned while seated with the head in a natural position, their teeth in slight contact, their lips closed and relaxed and their eyes open. Images were stored in the Digital Imaging and Communications in Medicine (DICOM) file format.

All data were imported into the surgical planning software (IPS CaseDesigner^®^; version 2.1.4.4; KLS Martin Group, Tuttlingen, Germany). All virtual osteotomies were implemented using the same procedure, and the bimaxillary orthognathic surgery was later performed in our clinic. The maxillary displacement simulation was based on a virtual Le Fort I osteotomy. The displacement of the mandible was performed according to the Obwegeser/Dal Pont osteotomy technique. Subsequently, fully automated soft-tissue simulation was performed in the VSP system IPS CaseDesigner^®^ and was exported as a STL file.

Four months after surgery, a 3D face scan of every patient was performed with a 3D camera (3dMDTrio.t System; 3dMD Limited; Brentford, London, United Kingdom) with the following parameters: capture speed, ~1.5 milliseconds; subject capture, 200° face capture (ear-to-ear); configuration, 3 modular units of 9 machine vision cameras and an industrial-grade flash system synchronized in a single capture; geometry accuracy, <0.2 mm root mean square (RMS). The scan was also saved as a STL file.

### 2.3. Validation of Soft-Tissue Simulation

**Figure 1:**
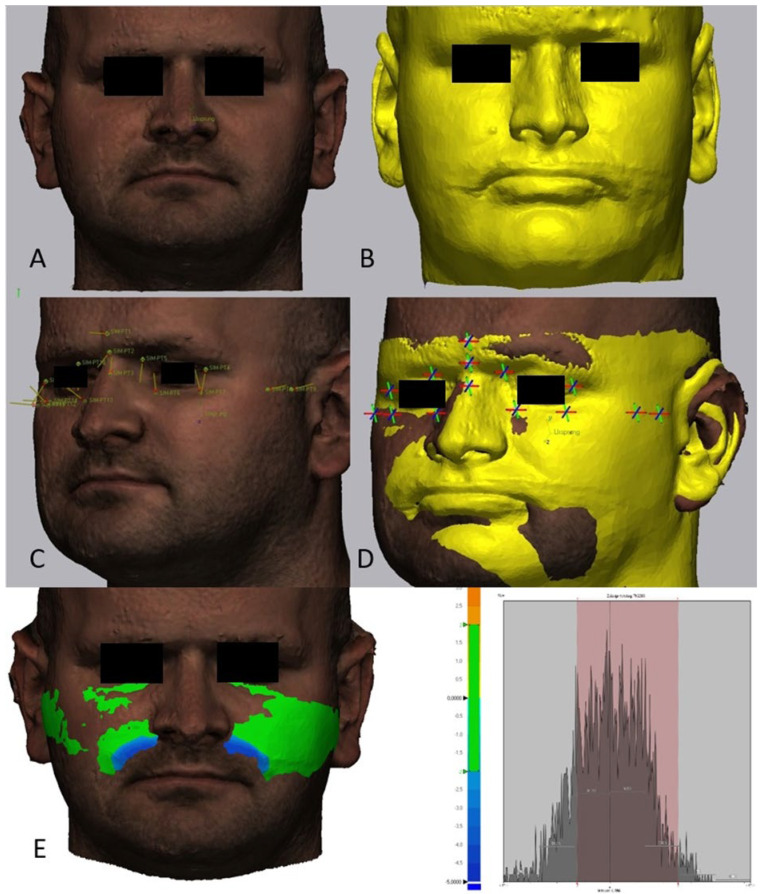
(**A**): Postoperative 3D scan, (**B**): Preoperative simulation of the postoperative soft-tissue (IPS CaseDesigner^®^), (**C**,**D**): RPS-Alignment, using 15 pairs of reference points in the nasal, periorbital, and zygomatic area of the face, (**E**): An example of the surface congruence for the anatomic area (upper cheek) illustrated with a colour-coded error map.

In order to measure the discrepancy between the soft-tissue simulation and the postsurgical 3D-Scan both STL files were implemented in the engineering software Geomagic Control X^TM^ (Version 2022.0.1, 3D Systems Inc., Rock Hill, SC, USA). Initially, the postoperative 3D scan was superimposed on the preoperative simulation of the soft-tissue as a ‘basic alignment’ (no reference points), as required by the software. Then, a RPS (reference point system)-alignment on the cranial part of the face, where no changes were expected due to the orthognathic surgery, was performed. Altogether, 15 pairs of points were positioned on both surfaces: 3 points (the glabella, nasion, and cranial part of the nose), mainly for the determination in the anterior-posterior dimension (x-axis); 4 points in the area of the zygomatic arches, mainly for determination in the lateral dimension (y-axis); and 8 points in the area of the inferior and superior medial and lateral orbital rim, for determination in the cranial-caudal (z-axis) and the lateral dimension (y-axis). The alignment was accepted if the corresponding deviations were <0.1 mm (Figure 1).

**Figure 2:**
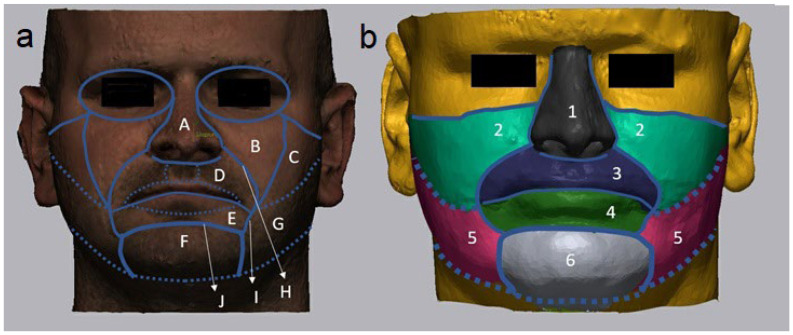
(**a**): Facial aesthetic units based on Fattahi et al. A: nose; B: cheek unit (medial subunit); C: cheek unit (zygomatic subunit); D: upper lip unit (philtrum + lateral + mucosal subunit); E: lower lip unit (central + mucosal subunit); F: mental unit; G: cheek unit (lateral + buccal subunit); H: melolabial fold; I: labiomandibular fold; J: mentolabial fold. (**b**): Segmentation of the soft-tissue simulation. 1: nose; 2: upper cheek (B+C); 3: upper lip (D); 4: lower lip (E); 5: lower cheek (G); 6: chin (F); 3+4: both lips; 1+2+3+4+5+6: face total.

Then, the quality of the soft-tissue simulation was validated with the help of a distance map. This distance map was calculated using the inter-surface distance algorithm between the preoperative simulation of the soft-tissue and the actual scan of the postoperative soft-tissue surface. To improve the validation, scattered radiation artifacts were omitted. Differences were visualized using a colour scale image indicating the magnitude of the difference, and the percentage of surface congruence was calculated (IO% values). After these results were obtained for the entire face, the region of interest was limited to 7 specific anatomic areas (nose, upper cheek, lower cheek, upper lip, lower lip, both lips, and chin), considering the facial aesthetic units (Figure 2). All the measurements were performed by the same researcher, who was experienced and trained in using the software.

The influence of a patient’s initial angle class, the magnitude of the displacement distance, the anterior bite situation, age, and gender were independently assessed to explain possible discrepancies. 

### 2.4. Statistical Analysis

Statistical analyses were performed with SPSS (version 25.0, IBM Corp., Armonk, NY, USA). All measured values were tested for the normal distribution by using the Shapiro-Wilk test. A multi-variate analysis (general linear model) assessed the effect of the independent parameters (angle class, total displacement distance, initial anterior bite situation, age, and gender) on the percentage of surface-congruence (IO%) of the different anatomic segments of the face (Table 2). The cut-off-value for significance was *p* < 0.05.

## 3. Results

The postoperative 3D scan of the facial soft-tissue was compared to the preoperative simulation regarding the percentage of adequate overlaid surface (±2 mm) (IO%), the mean absolute discrepancies (MTL) in mm, the unsigned mean absolute discrepancies (RMS) in mm and the standard deviation (SD) in mm.

**Figure 3:**
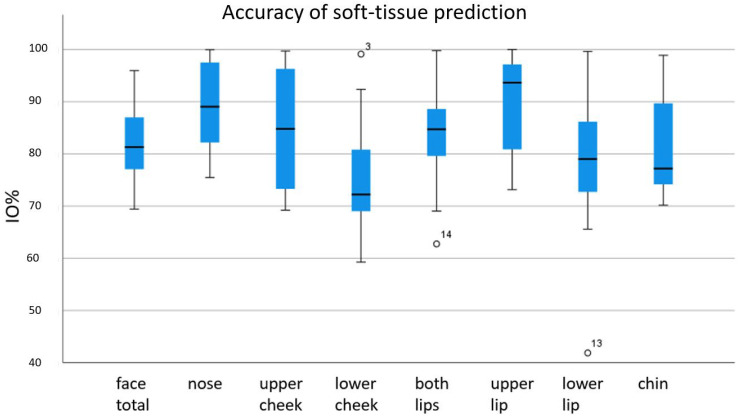
Percentage of correctly predicted soft-tissue: Surface congruence ± 2 mm. °: moderate outlier: 1.5–3.0 × IQR (interquartile range).

The values reached for IO% regarding the different anatomic areas of the face are presented in a box plot diagram (Figure 3). Values from 69.4% to 96.0% occurred for the face in total, 75.5% to 100% for the nose, 69.2% to 99.7% for the upper cheek, 59.3% to 99.1% for the lower cheek, 73.2% to 100% for the upper lip, 41.9% to 99.6% for the lower lip, 62.8% to 99.8% for both lips, and 70.2% to 98.9% for the chin area.

**Figure 4:**
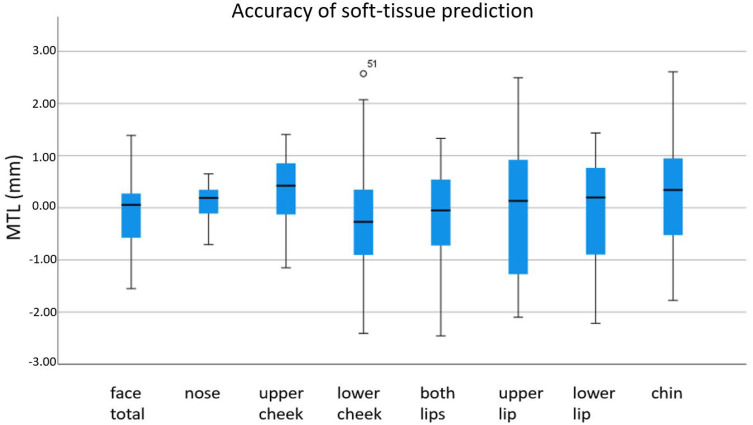
Mean absolute discrepancy between postoperative 3D scan and preoperative soft-tissue simulation. °: moderate outlier: 1.5–3.0 × IQR (interquartile range).

The mean discrepancies (MTL) in mm between the preoperative simulation and the postoperative scan are shown in Figure 4. Mean discrepancies reached values from −1.5 to 1.4 mm for the total face, −0.7 to 0.7 mm for the nose, −1.1 to 1.4 mm for the upper cheek, −2.4 to 2.6 mm for the lower cheek, −2.5 to 1.3 mm for the upper lip, −2.1 to 2.5 mm for the lower lip, −2.2 to 1.4 mm for both lips, and −1.8 to 2.6 mm for the chin area.

**Figure 5:**
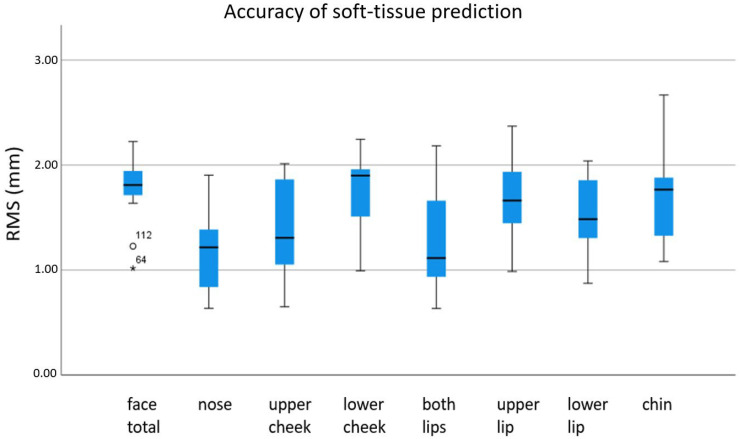
Unsigned mean absolute discrepancy between postoperative 3D scan and preoperative soft-tissue simulation. °: moderate outlier: 1.5–3.0 × IQR (interquartile range); *: extreme outlier: >3.0 × IQR.

**Figure 6:**
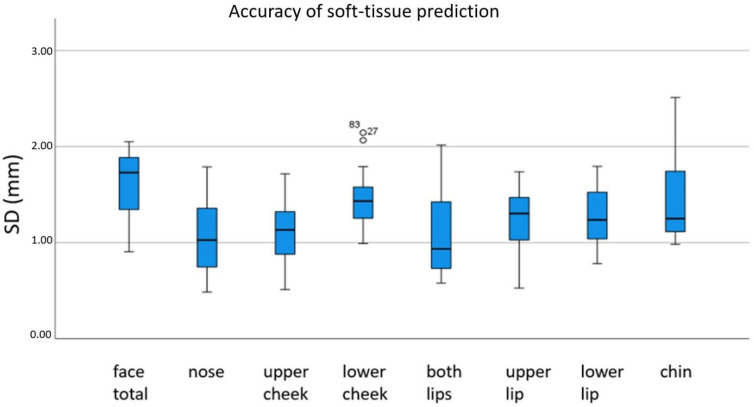
Standard deviation between postoperative 3D scan and preoperative soft-tissue simulation. °: moderate outlier: 1.5 – 3.0 × IQR (interquartile range).

The unsigned mean absolute discrepancies (RMS) in mm between the preoperative simulation and the postoperative scan are shown in Figure 5. The values for standard deviations are presented in Figure 6.

The influence of the independent parameters angle class, displacement distance, bite situation, age, and gender on IO% of the different regions of the face are shown in Table 2. Thereby, no significant influence could be revealed (*p*-values from 0.060 to 0.973).

## 4. Discussion

To our knowledge, this is the first study to analyse the predictability of soft-tissue changes using the VSP program IPS CaseDesigner^®^ in patients undergoing bimaxillary orthognathic surgery. 

Different surgical programs with soft-tissue simulation tools are available for 3D planning of orthognathic surgery such as Quick Ceph (Quick Ceph Systems Inc., San Diego, CA, USA), Dentofacial Planner (Dentofacial Software, Toronto, ON, Canada), ProPlan CMF (Materialise NV, Leuven, Belgium) and, the one most commonly discussed in the literature, Dolphin Imaging (Dolphin Imaging & Management Solutions, Chatsworth, CA, USA). The existence of numerous variables complicates surgical soft-tissue prediction. Applying a specific algorithm enables VSP systems to perform a soft-tissue prediction after a simulated osteotomy [2,6,11]. Modabber et al. stated, ‘the software differs with regard to its soft-tissue prediction according to the underlying physical model. Sparse models require landmarking and rely on interpolation between points, whereas other programs use dense volumetric models such as finite element, mass spring or tensor models, which need a volumetric tetrahedral mesh containing all the facial tissues [1,12]. In 3D prediction methods, it is impossible to predict soft-tissue changes following skeletal tissue by direct formulation or continuous equations and soft-to-hard tissue ratios because of different geometric complexities [10]. Different methods have been developed for this purpose, including purely geometrical models, multilayer mass-spring models (MSMs), finite element models (FEMs), and mass tensor models (MTMs) [13].

According to the developer, KLS Martin Group, the soft-tissue prediction of IPS CaseDesigner^®^ is based on the usage of tetrahedral MTMs [14]. MTM was introduced in the year 2000 by Cotin et al. [15] and Schwatz et al. [16]. Mollemans et al. described MTM as a mixture of FEM and MSM, combining the easy architecture of MSM with the biomechanical relevance of FEM. To predict the changes in the soft-tissue due to the orthognathic surgery, bone-related movements have to be mapped to the tetrahedral soft-tissue model. Therefore, the soft-tissue model is categorized into join points and free points. Join points are directly connected to the bone and highly likely equal the skeletal displacement; whereas, the movement of free points depends solely on the resulting elastic force that exists in these points and, therefore, on the characteristics of the specific tissue. Moreover, by combining the original MTM with a dynamic threshold to stop the iterations early, the simulation time can be considerably reduced [13].

VSP programs are said to have a soft-tissue prediction error of <2 mm. It is controversial whether this deviation has clinical relevance. It can be assumed, that those inaccuracies lie within the spectrum of reported and clinically expected bony discrepancies in orthognathic surgery [17,18,19,20,21]. Studies revealed outcome inaccuracies of 0.99 or 1.17 mm between planned and performed mono- and bi-maxillary orthognathic surgeries [3,12,22]. Moreover, according to Kaipatur et al., differences in the human face not larger than 2 mm are not noticeable to the human eye [23]. Thus, many researchers in the literature remark that prediction errors not exceeding 3 mm are not clinically significant [10,24,25,26]. That is why, in this study, discrepancies between the actual postoperative soft-tissue surface and the preoperative simulation of ±2 mm defined the scope of a correct prediction.

Moreover, the accuracy of data acquisition warrants discussion. According to Liang et al., with CBCT scanning, an image of a patient’s bone and soft-tissue can be acquired with an accuracy of 0.28 mm compared with the gold standard, laser surface scanning [27]. As outlined by Liebregts, CBCT imaging has advantages compared with multi-slice CT imaging. Advantages include a lower radiation dose, lower costs, and the possibility of scanning while the patient is seated [6]. The direct availability and ability to scan while the patient is seated allow for direct instructions from the maxillofacial surgeon to ensure a correct natural head position and relaxed lips with the proper occlusion. The postoperative scan was performed in the exact same position. A natural head position is important because an excessive head tilt or flexion can distort the tissue. Most importantly, the patient should relax the lips to avoid the muscle hyperfunction of grimacing during scanning. A standardized scanning protocol is essential to avoid the influence of variables. Other disadvantages include radiation artifacts from the cone radiation beam, which must be removed by the engineering software Control X^TM^, the absence of comparable Hounsfield units and different soft-tissue densities [6].

While CBCT is mandatory for surgical planning and prediction of changes, it is not necessary for postoperative control. Instead of a second CBCT for postoperative data acquisition, the authors therefore decided to use a 3D scan. The 3D scan, as it was performed, had a higher resolution and avoided unnecessary radiation. Using different image acquiring techniques is a certain weakness in study design. Nevertheless, we do not consider the technical differences to be so drastic as to justify additional radiation exposure to the patient for study purposes. The correct moment for the 3D scan for postoperative data acquisition is undetermined, since the time frame found in the literature is relatively wide [11]. In this study, all postsurgical scans were performed at least 4 months after surgery to ensure a minimization of any oedema of the soft-tissue in order to produce a realistic result. The large time span of postsurgical 3D scans could be one limitation of this study. Further movements due to ossification, muscle line changes, or those caused by orthodontic treatment would not be depicted [2]. Nadjmi et al. recommend that postsurgical outcomes should be recorded at the same time for all patients, after a complete decline of swelling but before orthodontic treatment [7,11]. Moreover, the preoperative CBCT and the postoperative 3D scan were both performed with the multiband appliance in situ to produce comparable results, as far as the position of the lips is concerned. However, it is sometimes difficult to determine the exact time, according to Nadjmi et al., as there is high inter-individual variability in the reduction of swelling, and orthodontic postoperative treatment often starts early [7]. Additionally, in a few cases, it might be difficult to differentiate swelling from the regular soft-tissue changes caused by surgery.

Regarding the entire face, accuracy values (IO%) from 69.4% to 96.0% were reached, with mean discrepancy values from −1.5 to 1.4 mm. These results are comparable with other published findings. Bianchi et al. and Marchetti et al. reported a reliable simulation outcome for the entire face, with average absolute errors of 0.94 and 0.75 mm, respectively, and a high accuracy with an error lower than the 2 mm tolerance level (86.8% and 91%, respectively) [28,29]. Mollemans et al. reported an average median distance measuring 0.60 mm, and the 90th percentile was smaller than 1.5 mm for measurements of the entire face. They noted the greatest deviations in the lip and the chin areas [13]. An even higher accuracy of soft-tissue simulation in orthognathic patients was achieved by Schendel et al. in their study using the VSP system Vultus software (3dMD, Atlanta, GA). They evaluated 23 patients (mono- and bimaxillary) and obtained an overall accuracy of 0.27 mm, with the largest deviation in the perioral region [6,30].

In this study, the facial region that was best predicted by the VSP system IPS CaseDesigner^®^ was that of the nose, including the entire soft-tissue of the nose and a small rim of the paranasal tissue. Thereby, a prediction accuracy (IO%) of 75.5% to 100% was achieved, with mean deviations smaller than ±1 mm. Differences occurred in the lower area of the nose, more precisely at the nasal tip and the nasal base at the transition to the upper lip; whereas, the cranial part of the nose was predicted nearly 100% correctly in all of the cases. This result is not surprising since minimal changes to the nose are expected when a bimaxillary orthognathic surgery is performed. Peterman et al. also reported that the pronasal point with deviations of 0.5 mm was the most accurate prediction; whereas, the lip predictions were the most inaccurate. It should be mentioned that in their study, the VSP system Dolphin Imaging was used in the analysis, and bimaxillary osteotomies were performed only for angle class III patients [9].

A further study, which evaluated the soft-tissue predictability of two different VSP systems (Dolphin Imaging and ProPlan), showed a postoperative inaccuracy of approximately <2 mm for the root mean square distance for the nose and the paranasal region [31]. The authors attribute this discrepancy to the subnasal point, which changes more than the upper lip when a Le Fort I osteotomy is performed. In this context, DeSesa et al. reported a change in the subnasal point of 1.3 ± 1.8 mm after a 5.8 mm displacement [32]. The abovementioned studies demonstrated soft-tissue simulations comparable to the results of our study. In a further study by Liebregts et al., the subnasal point could be precisely predicted with a mean absolute difference of 1.1 mm. However, it must be taken into consideration that only single-jaw orthognathic surgery (mandible only) was performed in the abovementioned study, so a small change in the subnasal region was expected anyway [6]. Moreover, Ahmad Akhoundi et al. also evaluated the soft-tissue predictability of the VSP system Dolphin Imaging. They stated that the nasal tip was best predictable and had the highest reliability [33].

Regarding the perioral region, in this study, the upper lip was more precisely predicted than the lower lip, which achieved the lowest values for IO%. Peterman et al. explained that the inaccuracy of lower lip prediction was due to both the effect of the anterior tooth position on the lower lip and the muscle tone of the perioral muscles [9]. Furthermore, Maal et al. came to similar conclusions by examining a different VSP system. The region with the lowest accuracy was the lower lip area. The mean absolute 90th percentile of the deviation in the lower lip area was 2.5 mm in their study [34]. Of course, it cannot be ruled out that the low values of prediction accuracy in the region of the lower lip were also caused by movements made by the patient during the CBCT or the 3D scan, although all patients received clear instructions, and the position of the patient’s head was controlled by the same operator every time. By contrast, Demirsoy et al. obtained the least accurate predictions, with a mean difference exceeding 3 mm, in the upper lip area using the VSP system SimPlant master software [10].

In this study, the chin region was predicted slightly more precisely than the lower lip region but not as precisely as the upper lip region. This corresponds with the findings of other studies, for example, that of Liebregts et al. [6]. According to Modabber et al., the high error rate in the chin area in their study was caused by the fact that impaction of the maxilla is generally performed together with mandibular displacement [35,36] and the subsequent autorotation is not taken into account in the software algorithms [12]. The displacement and autorotation of the mandible can also be seen as the main reasons why, in this study, the cranial cheek region was predicted more precisely than the lower cheek area. Finally, the changes in soft-tissue in the cranial part of the face are only caused by the displacement of the maxilla; whereas, the soft-tissue in the caudal part of the face follows the displacement of the mandible and partially follows that of the maxilla, as well as autorotation. This multifactorial influence makes it even more difficult to develop an algorithm for precise prediction. This proves the importance of the bony displacement for the predictability of the soft-tissue. Insofar, it can be critically noted that in this study no additional examination of the correct implementation of the planning with regard to bony displacements took place. However, as mentioned above, studies have already demonstrated the high accuracy of the implementation of digital planning in orthognathic surgery. Due to the already existing studies [17,18,19,22], which only suggests a low inaccuracy of 0.99 to 1.17 mm, and the fact that an additional CBCT would be necessary, such a test was therefore deliberately omitted in the interest of patient safety. Several additional factors that could influence the operative soft-tissue are discussed in the literature. Age, gender, and race are said to have an influence on the changes in the facial soft-tissue when orthognathic surgery is performed [10]. Other possible influential factors that have been previously described are variation in the thickness of soft-tissues, its swelling after surgery, weight gain or loss in the postoperative period, the presence of rigid orthodontic appliances preoperatively, and dental changes owing to orthodontic treatment after surgery [6]. The VSP systems undergo continuous development and perfection of the underlying algorithms, taking the abovementioned parameters increasingly into account. In this study, the tested independent parameters (angle class, displacement distance, bite situation, age, and gender) had no influence on the predictability of the postsurgical soft-tissue (Table 2). Moreover, in other current studies, no correlation was found between the error in soft-tissue simulation and age, gender, displacement distance, or the amount of mandibular rotation [6].

## 5. Conclusions

The primary intention of the study was to test the planning software, in terms of accuracy of the software’s prediction of the soft-tissue profile. In particular, this was to be done in a manner and by means that represented everyday clinical practice and did not impose additional radiation exposure on the patient. Finally, in due consideration of the limitations of this study, it can be concluded that the VSP system IPS CaseDesigner^®^ predicts the patient’s operative soft-tissue accurately. There are still single factors that are difficult to control and predict that affect the soft-tissue response in orthognathic surgery. It should be a duty to inform patients before surgery about the fact that soft-tissue predictions might only guide and not necessarily demonstrate the actual postoperative result [1]. A continuous improvement of the underlying algorithm is needed for a precise prediction of soft-tissue changes, especially for the lower part of the face.

## Figures and Tables

**Table 1:** Patient’s characteristics.

	Angle Class II/III	Displacement Distance <5 mm/>5 mm	Anterior Bite Situation Deep/Neutral/Open	Age <30a/30–40a/>40a	Gender Male/Female
Number	9/11	10/10	8/7/5	11/5/4	10/10

**Table 2:** Influence of the parameters (angle class, displacement distance, bite situation, age, and gender) on the IO%> Statistical significance was considered at *p* < 0.05.

	Face Total	Nose	Upper Cheek	Lower Cheek	Upper Lip	Lower Lip	Both Lips	Chin
Parameter	*p*	*p*	*p*	*p*	*p*	*p*	*p*	*p*
Angle class	0.428	0.165	0.648	0.977	0.899	0.383	0.463	0.132
Displacement distance	0.097	0.227	0.335	0.063	0.323	0.170	0.376	0.640
Bite situation	0.761	0.149	0.403	0.072	0.408	0.866	0.604	0.497
Age	0.973	0.253	0.362	0.902	0.648	0.966	0.870	0.717
Gender	0.644	0.060	0.257	0.127	0.543	0.443	0.629	0.502
